# Addressing Unmet PrEP Needs in Women: Impact of a Laboratory-Driven Protocol at an Urban, Essential Hospital

**DOI:** 10.1093/ofid/ofae056

**Published:** 2024-02-01

**Authors:** Jessica Stewart, Glorimar Ruiz-Mercado, Heather Sperring, Cassandra M Pierre, Sabrina A Assoumou, Jessica L Taylor

**Affiliations:** Section of Infectious Disease, Boston University Chobanian and Avedisian School of Medicine and Boston Medical Center, Boston, Massachusetts, USA; Section of Infectious Disease, Boston University Chobanian and Avedisian School of Medicine and Boston Medical Center, Boston, Massachusetts, USA; Grayken Center for Addiction, Boston Medical Center, Boston, Massachusetts, USA; Section of Infectious Disease, Boston University Chobanian and Avedisian School of Medicine and Boston Medical Center, Boston, Massachusetts, USA; Section of Infectious Disease, Boston University Chobanian and Avedisian School of Medicine and Boston Medical Center, Boston, Massachusetts, USA; Section of Infectious Disease, Boston University Chobanian and Avedisian School of Medicine and Boston Medical Center, Boston, Massachusetts, USA; Section of General Internal Medicine, Boston University Chobanian and Avedisian School of Medicine and Boston Medical Center, Boston, Massachusetts, USA; Grayken Center for Addiction, Boston Medical Center, Boston, Massachusetts, USA

**Keywords:** care disparities, HIV pre-exposure prophylaxis (PrEP), HIV prevention, sexually transmitted infection, women’s health

## Abstract

**Background:**

HIV pre-exposure prophylaxis (PrEP) uptake in women remains low. We developed a laboratory result–driven protocol to link women with a positive bacterial sexually transmitted infection (STI) to HIV PrEP at an urban safety-net hospital.

**Methods:**

Electronic health records of women with positive chlamydia, gonorrhea, and/or syphilis tests were reviewed, and those eligible for PrEP were referred for direct or primary care provider-driven outreach. We assessed the proportion of women with STIs who received PrEP offers, acceptance, and prescriptions before (July 1, 2018–December 31, 2018) and after (January 1, 2019–June 30, 2020) implementation to evaluate changes in the delivery of key elements of the PrEP care cascade (ie, PrEP offers, acceptance, and prescribing) for women with STIs after protocol implementation.

**Results:**

The proportion of women who received PrEP offers increased from 7.6% to 17.6% (*P* < .001). After multivariable adjustment, only the postintervention period was associated with PrEP offers (odds ratio [OR], 2.49; 95% CI, 1.68–3.68). In subgroup analyses, PrEP offers increased significantly among non-Hispanic Black (OR, 2.75; 95% CI, 1.65–4.58) and Hispanic (OR, 5.34; 95% CI, 1.77–16.11) women but not among non-Hispanic White women (OR, 1.49; 95% CI, 0.54–4.05). Significant changes in PrEP acceptance and prescriptions were not observed in the sample overall.

**Conclusions:**

A laboratory result–driven protocol was associated with a significant increase in PrEP offers to Black and Hispanic women with STI. These results provide concrete suggestions for health systems seeking to increase PrEP access and equity among women.

In 2019, nearly 7000 women were diagnosed with HIV in the United States, where structural racism continues to drive profound inequities. Although Black women only comprise 13% of the female population, 58% of women diagnosed with HIV are Black; an additional 17% of women newly diagnosed with HIV are Hispanic [1]. This high HIV transmission occurs against a backdrop of inadequate HIV prevention services for women, including gaps in access and uptake to HIV pre-exposure prophylaxis (PrEP) [2–6].

HIV PrEP with daily oral tenofovir disoproxil fumarate/emtricitabine (TDF/F) is a powerful tool for HIV prevention, reducing HIV incidence by 99% in people with sexual risk and up to 74% in people with injection-related risk and very high adherence [7–9]. Long-acting injectable cabotegravir provides PrEP with even higher HIV prevention efficacy [10].

However, PrEP uptake in women remains inadequate [3, 11]. In 2019, just 7.4% of people on PrEP were women, and the PrEP-to-need ratio (ie, the number of people on PrEP compared with the number of people newly diagnosed with HIV) was just 2.34 in women compared with 6.86 in men [12]. The available data from outpatient settings where primary care providers (PCPs) are a main source of referrals to PrEP indicate that only 4.9% of PrEP-linked patients had female sex listed in the electronic health record (EHR) [13]. Low rates of PrEP prescribing in women suggest that even those with straightforward PrEP indications such as bacterial sexually transmitted infection (STI) are not receiving PrEP. Uptake in Black and Hispanic women remains alarmingly low due to barriers at the individual, network, health care, and structural levels [14].

The overall lack of PrEP delivery in primary care and other settings has spurred interest in the use of EHR algorithms and machine learning to identify PrEP candidates [15–22]. These algorithms, which are systems-level interventions that do not rely on individual patients or providers, promise substantial benefit in identifying women at high risk of HIV who are not identified for HIV prevention services through regular clinical practice. However, the informatics infrastructure required to implement complex EHR algorithms is not yet available to all health systems, including safety-net settings serving patients with high social vulnerability.

In January 2019, our team developed a laboratory result–driven protocol to identify PrEP-eligible women with a bacterial STI at higher risk for HIV. The goal of the current study was to evaluate changes in PrEP offers, a key early element of the PrEP cascade, after implementation of the protocol. Secondary aims included evaluating changes in PrEP acceptance and prescribing. To assess for equity in the intervention, we additionally evaluated differential changes by race and ethnicity.

## METHODS

### Setting

Boston Medical Center (BMC), affiliated with Boston University in Suffolk County, Massachusetts, is the largest safety-net hospital in New England [23]. In the 18 months before February 2022, BMC served a diverse population of >270 000 patients, 32% of whom were non-Hispanic Black, 24% of whom were Hispanic (any race), and 30% of whom spoke a non-English primary language. Prevalent structural barriers include poverty, unemployment, unstable housing/homelessness, and food insecurity [24].

Suffolk County is one of 48 priority counties in the federal “Ending the HIV Epidemic: A Plan for America” initiative and the location of one-quarter of Massachusetts HIV diagnoses [25, 26]. At BMC, women accounted for 36% of new HIV infections in 2022, a significantly higher proportion than the national average of 19% [27], and diagnoses were concentrated in Black women (81%).

Funding from the Massachusetts Department of Public Health supports a dedicated STI clinic as well as a hospital-wide HIV PrEP team, which includes a PrEP Quality Manager (QM), a part-time Infectious Disease physician, a part-time General Internal Medicine physician, and 2 PrEP Navigators.

The PrEP team provides resources to support PrEP initiation across departments, including educational sessions, EHR order sets and note templates, and clinical consultation. Clinicians who are not comfortable starting PrEP can refer their patients for PrEP consult visits in the General Internal Medicine (GIM), Infectious Disease, and STI clinics or within subspecialty addiction settings. Additionally, patients can be referred for PrEP case management (eg, medication cost assistance and navigation support). Before January 2019, patients with STI were not systematically referred for PrEP consultation; PrEP initiation or referral was dependent upon the ordering provider.

### Study Design and Intervention

We conducted a retrospective pre/postintervention study. On January 1, 2019, the HIV PrEP team implemented a daily, institution-wide report (“STI report”) to aggregate positive test results for chlamydia, gonorrhea, and syphilis (syphilis immunoglobulin [Ig]G/IgM antibodies, rapid plasma regain [RPR], and *Treponema pallidum* particle agglutination assay [TPPA]) [28]. Using Centers for Disease Control and Prevention (CDC) criteria, the QM reviewed the EHR of patients listed on the report to determine if individuals were potentially eligible for PrEP. Outreach was conducted with each unique STI diagnosis. The majority of participants received a single outreach attempt; on a case-by-case basis (eg, provider asked for help because they could not reach the patient to disclose the positive STI result), additional calls were made. Positive gonorrhea and syphilis results were prioritized; the charts of patients with chlamydia were reviewed only based on capacity due to very high volume.

Potential candidates were referred to the BMC PrEP program via 1 of 2 pathways. First, if patients had a BMC PCP (ie, at least 1 visit in the past 3 years to a BMC GIM, Family Medicine, Geriatrics, or Pediatric PCP), the QM sent the PCP an EHR message alerting them to the patient's potential PrEP eligibility and institutional supports [28]. If the PCP did not respond within 5 days, a PrEP Navigator called the patient and offered HIV risk assessment and PrEP in the BMC STI clinic. Second, patients without a BMC PCP were contacted directly by a PrEP Navigator and offered the same resources.

### Data Collection

We used the STI report and EHR data to identify cis- and transgender women aged 18 years and older with positive STI result(s) in the 6 months before (July 1, 2018–December 31, 2018) and 18 months after (January 1, 2019–June 30, 2020) the intervention.

Women were identified by either female administrative sex in the EHR, and/or female gender identity in instances where the latter was available. Individuals with known HIV infection at the time of STI diagnosis and those with an estimated glomerular filtration rate <60 mL/min were excluded due to clinical ineligibility for approved PrEP medications during the study period.

Demographic variables abstracted from the STI report, the EHR, and existing clinical databases included age, race/ethnicity, spoken language, and Social Vulnerability Index (SVI). The SVI uses US census data to identify communities at increased risk during hazardous events and public health emergencies. SVI is presented as a percentile ranking indicating the proportion of areas with lower social vulnerability [29]. For example, an SVI of 0.5 indicates that 50% of areas have lower social vulnerability. Clinical variables included STI test date, order location, result, treatment, and date of most recent HIV testing. To evaluate the outcome of HIV seroconversion after STI, we abstracted HIV result data from July 1, 2018, through July 31, 2021.

We also abstracted key elements of the PrEP care cascade. PrEP offers were defined as contact made with the patient to offer PrEP. PrEP acceptance was defined as verbal patient agreement documented in the EHR to initiate PrEP. PrEP prescriptions were defined as an EHR prescription for tenofovir disoproxil fumarate/emtricitabine (TDF/FTC), the only Food and Drug Administration–approved PrEP medication for people at risk for HIV through vaginal sex during the study period.

### Data Analysis

We used descriptive statistics to characterize the study population. Participants were included in both the pre- and postintervention periods if they had STIs during both periods. Participants with >1 STI during the pre and/or post period were only included once within each time period using the date of their first positive STI test or tests (if positive for 2 or more infections concurrently).

The primary outcome was PrEP offers. We used mixed-effects logistic regression to determine associations between covariates and the primary outcome. Variables were tested against one another for collinearity using Pearson's correlation coefficient. If correlation was found between 2 variables, the variable with the stronger significance was retained in the final model. Variables on the causal pathway were excluded from the final multivariable model. Odds ratios and 95% confidence intervals were reported. In secondary analyses, we used mixed-effects logistic regression to calculate the association between our intervention and other key steps in the PrEP care cascade: acceptance and prescriptions. Offers, acceptance, and prescriptions were included if they occurred within 6 months of the index STI.

To evaluate for equity in the intervention, we created 2 separate final adjusted models for the primary outcome and the other PrEP care cascade steps, 1 stratified by race/ethnicity and 1 stratified by type of STI. First, univariate analyses using the chi-square and Fisher exact tests were used to evaluate significant differences among non-Hispanic Black, non-Hispanic White, and Hispanic patients to determine variables to include in the multivariate models. If correlation was found between 2 variables, the variable with the stronger significance was retained in the final model. Because women with gonorrhea and syphilis infection were prioritized for the intervention, we also ran mixed-effects logistic regression models stratified by gonorrhea/syphilis vs chlamydia infection. For these analyses, women with concurrent gonorrhea and chlamydia or concurrent syphilis and chlamydia were categorized as having gonorrhea and syphilis, respectively. Odds ratios, 95% confidence intervals, and *P* values (using a significance value of .05) were reported.

All analyses were done in SAS, version 9.4 (Cary, NC, USA).

### Patient Consent

This study was approved by the Boston University Medical Campus Institutional Review Board and was granted exempt status (H-40621). This study did not include factors necessitating patient consent.

## RESULTS

### Participant Characteristics

Overall, 1549 unique women age 18 years and older had a positive STI during the study period; 110 were excluded due to PrEP ineligibility (chronic kidney disease, 46; known HIV infection, 62; concurrent incident HIV, 2), leaving 1439 unique patients in the study sample. The pre-intervention period included 421 unique women, and the postintervention period included 1086 women. Sixty-eight women appeared in both study periods (Figure 2).

**Figure 1.**
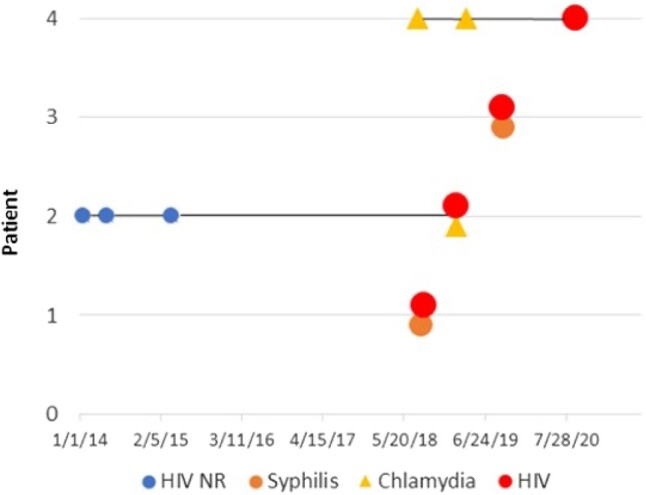
HIV seroconversions among women with STI. Abbreviation: NR, non-reactive.

**Figure 2.**
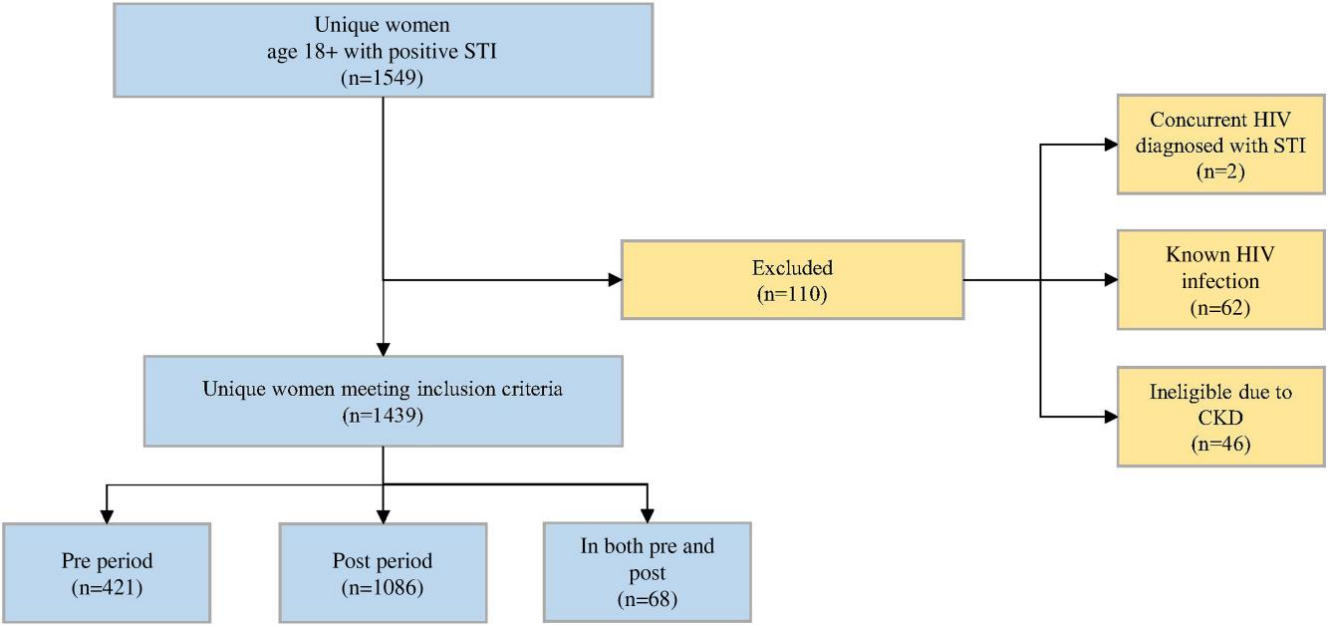
Women with STI at an urban safety-net hospital, Boston, Massachusetts, July 1, 2018–June 30, 2020. Abbreviations: CKD, chronic kidney disease; STI, sexually transmitted infection.

Participants’ mean age (SD) was 30.4 (14.9) years, 850 (59.1%) were non-Hispanic Black, 327 (22.7%) were Hispanic (all races), and 132 (9.2%) were non-Hispanic White. The mean SVI (SD) was 0.697 (0.247), indicating that participants lived in areas with higher social vulnerability than 70% of other areas (Table 1).

**Table 1.** Characteristics of Women With Sexually Transmitted Infection at an Urban Safety-Net Hospital, July 2018–June 2020

	Overall (n = 1439)	Pre-intervention (n = 421)	Postintervention (n = 1086)	*P* Value
Age, mean (SD), y	30.4 (14.9)	29.0 (13.5)	31.0 (15.5)	.0122
Race/ethnicity, No. (%)				.628
Black, non-Hispanic	850 (59.1)	256 (60.8)	639 (58.8)	…
Hispanic	327 (22.7)	93 (22.0)	247 (22.7)	…
White, non-Hispanic	132 (9.2)	41 (9.7)	94 (8.7)	…
Other race, non-Hispanic	27 (1.9)	6 (1.4)	22 (2.0)	…
Unknown	103 (7.2)	25 (5.9)	84 (7.7)	…
Primary language, No. (%)				.743
English	1043 (72.5)	316 (75.1)	781 (71.9)	…
Spanish	157 (10.9)	42 (10.0)	120 (11.1)	…
Haitian Creole	106 (7.4)	28 (6.7)	82 (7.6)	…
Cape Verdean	77 (5.4)	22 (5.2)	58 (5.3)	…
Other	56 (3.9)	13 (3.1)	45 (4.1)	…
SVI, mean (SD)	0.697 (0.25)	0.692 (0.26)	0.701 (0.24)	.547
Tested at primary care,^a^ No. (%)				.473
Yes	519 (36.1)	148 (35.2)	406 (37.4)	…
No	911 (63.3)	269 (63.9)	675 (62.1)	…
Unknown	9 (0.6)	4 (1.0)	5 (0.5)	…
HIV concurrent test, No. (%)				.132
Yes	502 (34.9)	134 (31.8)	392 (36.1)	…
No	937 (65.1)	287 (68.2)	694 (63.9)	…

Abbeviations: STI, sexually transmitted infection; SVI, Social Vulnerability Index.

^a^Primary care sites include general internal medicine, family medicine, pediatrics, and geriatrics.

Approximately one-third (36.1%) of women with a positive STI had been tested at their primary care site. Participant characteristics were similar in the pre- and postintervention periods, though postintervention participants were slightly older (mean age, 31 vs 29 years; *P* = .0122) (Table 1).

### STI Testing & Results

Overall, 72.3% (n = 1040) had a positive chlamydia result, 18.1% (n = 184) had a reactive syphilis test, and 12.8% (n = 261) had a positive gonorrhea result at the time of their first observation. Forty-six patients had 2 or more positive STI results concurrently during their first observation in the pre or post period. Only approximately one-third (33.6%) were tested for HIV concurrent to the positive STI.

### PrEP Offers

The proportion of women with a positive STI who were offered PrEP increased from 7.6% pre-intervention to 17.6% postintervention (*P* < .001). In the pre-intervention period, outreach attempts to 33 unique patients yielded 32 PrEP offers, whereas in the postintervention period, outreach attempts to 256 unique patients yielded 191 PrEP offers.

In univariate analyses, other race, Haitian Creole primary language, and positive chlamydia result were negatively associated with PrEP offers. Positive gonorrhea, positive syphilis, and intervention period were significantly positively associated with PrEP offers (Table 2). Race/ethnicity and primary language were highly correlated; therefore only race/ethnicity was retained for the final model. Positive chlamydia, gonorrhea, and syphilis results were not included in the final model because they are on the causal pathway for PrEP offers. All other variables were retained in the final model due to either statistical significance or clinical significance. Intervention period was the only variable significantly associated with PrEP offers in the adjusted model.

**Table 2.** PrEP Offers to Women With STI at an Urban Safety-Net Hospital Before and After a Laboratory Result–Driven PrEP Intervention

	Not Offered PrEP (n = 1283), No. (%)	Offered PrEP (n = 224), No. (%)	Unadjusted OR (95% CI)	Adjusted OR (95% CI)
Age, mean (SD), y	30.1 (15.0)	32.6 (14.4)	1.01 (1.00–1.02)	1.01 (0.98–1.03)
Race/ethnicity, No. (%)				
Black, non-Hispanic	759 (59.2)	136 (60.7)	0.78 (0.49–1.26)	1.31 (0.80–2.17)
Hispanic	294 (22.9)	46 (20.5)	0.69 (0.40–1.18)	1.48 (0.85–2.58)
White, non-Hispanic	110 (8.6)	25 (11.2)	Ref.	Ref.
Other race, non-Hispanic	27 (2.1)	1 (0.45)	0.16 (0.02–1.26)	6.43 (0.83–50.04)
Unknown	93 (7.3)	16 (7.1)	0.76 (0.38–1.51)	1.39 (0.68–2.83)
Primary language, No. (%)				
English	922 (71.9)	175 (78.1)	Ref.	…
Spanish	142 (11.1)	20 (8.9)	0.74 (0.45–1.21)	…
Haitian Creole	100 (7.8)	10 (4.5)	0.53 (0.27–1.03)	…
Cape Verdean	71 (5.5)	9 (4.0)	0.67 (0.33–1.36)	…
Other	48 (3.7)	10 (4.5)	1.10 (0.54–2.22)	…
SVI, mean (SD)	0.701 (0.245)	0.686 (0.260)	0.84 (0.48–1.48)	1.14 (0.55–1.74)
Tested at primary care,^a^ No. (%)				
Yes	464 (36.2)	90 (40.2)	1.12 (0.89–1.60)	0.95 (0.69–1.30)
No	812 (63.3)	132 (58.9)	Ref.	Ref.
Unknown	7 (0.6)	2 (0.9)	1.76 (0.89–1.60)	0.54 (0.10–2.92)
HIV concurrent test, No. (%)				
Yes	431 (33.6)	95 (42.4)	1.46 (1.09–1.95)	0.75 (0.55–1.02)
No	852 (66.4)	129 (57.6)	Ref.	…
Positive STI result, No. (%)				
Chlamydia	986 (76.9)	94 (42.0)	0.22 (0.16–0.29)	…
Gonorrhea	111 (8.7)	85 (38.0)	6.46 (4.63–9.01)	…
Syphilis	210 (16.4)	70 (31.3)	2.32 (1.69–3.20)	…
Intervention period, No. (%)				
Pre-intervention	388 (30.2)	33 (14.7)	Ref.	Ref.
Postintervention	895 (69.8)	191 (85.3)	2.51 (1.70–3.70)	2.49 (1.68–3.68)

Abbreviations: PrEP, pre-exposure prophylaxis; SVI, Social Vulnerability Index.

^a^Primary care sites include general internal medicine, family medicine, pediatrics, and geriatrics.

### PrEP Care Cascade

Although PrEP offers increased, subsequent steps in the care cascade—PrEP acceptance and prescriptions—did not increase significantly (Table 3).

**Table 3.** PrEP Care Cascade for Women With STI at an Urban Safety-Net Hospital Before and After a Laboratory Result–Driven PrEP Intervention

	Overall (n = 1439)	Pre-intervention (n = 421)	Postintervention (n = 1086)	*P* Value
PrEP offered	223 (15.6)	32 (7.6)	191 (17.6)	<.001
PrEP accepted	67 (4.7)	12 (2.9)	55 (5.1)	.051
PrEP prescribed	38 (2.6)	6 (1.4)	32 (3.0)	.101

Abbreviations: PrEP, pre-exposure prophylaxis; STI, sexually transmitted infection.

### PrEP Equity

In univariate analysis stratified by race/ethnicity, significant differences were seen in age (mean age, 31.7 years for non-Hispanic Black patients, 28.7 years for non-Hispanic White patients, and 28.6 years for Hispanic patients; *P* = .003), language, SVI, and test order location. Age and lab order location were included in multivariate models.

In multivariate mixed-effects logistic regression models stratified by race/ethnicity, significant increases in the primary outcome of PrEP offers were observed among non-Hispanic Black and Hispanic women, but not among non-Hispanic White women (Table 4). Non-Hispanic Black women additionally had significant increases in PrEP acceptance that were not observed in the sample overall. No changes in PrEP prescriptions were observed (Table 4).

**Table 4.** Odds of PrEP Care Cascade Completion Pre- vs Postintervention by Race/Ethnicity

	Black, Non-Hispanic^a^ OR (95% CI)	*P* Value	White, Non-Hispanic^a^ OR (95% CI)	*P* Value	Hispanic^a^ OR (95% CI)	*P* Value
PrEP offered	2.75 (1.65–4.58)	<.0001	1.49 (0.54–4.05)	.440	5.34 (1.77–16.11)	.002
PrEP accepted	2.96 (1.14–7.68)	.025	0.30 (0.07–1.19)	.086	1.80 (0.96–3.41)	.069
PrEP prescribed	2.08 (0.70–6.14)	.187	0.73 (0.06–8.65)	.927	2.02 (0.84–4.88)	.118

Abbreviations: PrEP, pre-exposure prophylaxis; OR, odds ratio.

^a^Controlling for age and tested at primary care site (y/n).

Among women with positive gonorrhea and syphilis results, who were prioritized for the intervention, PrEP offers increased from 15.2% pre-intervention to 37.9% postintervention. Differences in PrEP acceptance and prescriptions were not observed (Table 5). Among women with chlamydia, PrEP offers also increased significantly, though to a lesser degree (Table 5).

**Table 5.** Odds of PrEP Care Cascade Completion Pre- vs Postintervention Among Women With Gonorrhea and Syphilis

	Gonorrhea+ and Syphilis+^a^ OR (95% CI)		Chlamydia+^a^	*P* Value
PrEP offered	3.80 (2.15–6.71)	<.001	2.19 (1.25–3.81)	.006
PrEP accepted	2.52 (1.04–6.14)	.041	1.38 (0.54–3.49)	.501
PrEP prescribed	2.68 (0.79–9.11)	.114	1.29 (0.35–4.82)	.702

Abbreviations: PrEP, pre-exposure prophylaxis; OR, odds ratio.

^a^Controlling for age and race/ethnicity.

### HIV Seroconversion

Four study participants were newly diagnosed with HIV (Figure 1). They included 3 cis-gender, heterosexual women and 1 transgender woman. One woman had noninjection cocaine and alcohol use disorders, and another had injection opioid and stimulant use disorders and a history of transactional sex. All women who seroconverted during the study were non-Hispanic Black.

Patient 1 was not screened for HIV at the time of secondary syphilis diagnosis and tested positive for HIV-1 1 month later (baseline HIV-1 RNA 12 392 copies/mL, CD4 443 cells/mm^3^). Patient 2 had 3 negative HIV tests and was subsequently diagnosed with chlamydia and HIV-1 concurrently (HIV-1 RNA 13 200 copies, CD4 416 cells/mm^3^). On the basis of chart review, she had not previously been offered or prescribed PrEP. Patient 3 was diagnosed with late latent syphilis of unknown duration and HIV-2 concurrently (HIV-2 RNA 13 copies, CD4 109 cells/mm^3^). Her HIV-2 infection was likely longstanding, reflecting a missed opportunity for earlier detection. Patient four was diagnosed with chlamydia twice, in both the pre- and poststudy periods. She was not tested for HIV at the time of chlamydia diagnoses and was not offered PrEP. She tested positive for HIV-1 26 months after her initial positive chlamydia test (HIV-1 RNA 1990 copies, CD4 318 cells/mm^3^).

## DISCUSSION

The current study describes a hospital-wide intervention to increase PrEP delivery to women with STIs. Though staffing limitations impacted intervention delivery, the intervention was nonetheless effective in increasing PrEP offers to eligible women with impacts that were most pronounced among Black and Hispanic women. Our study also incorporated the CDC SVI to assess social vulnerability, demonstrating intervention efficacy in increasing PrEP offers in a population with higher SVI—that is, greater social need—than 70% of those in other areas.

Our results build upon work demonstrating PrEP initiation among Black and Hispanic women receiving PrEP navigation services [30]. Our study adds to the literature in describing an intervention associated with increased PrEP offers to Black and Hispanic women and increased PrEP acceptance by non-Hispanic Black women, neither of which were observed in White women. Although this study was not designed to evaluate the mechanism of increased effectiveness in Black and Hispanic women, it is possible that the lack of effect in White women was due to the disproportionate impact STIs have on Black and Hispanic women, therefore limiting intervention delivery in White women. It is also possible that care was facilitated by a diverse, bilingual, and bicultural PrEP team. The mechanisms of efficacy among Black and Hispanic women warrant further investigation.

Importantly, although the rate of PrEP offers increased from 7.6% to 17.6%, study results indicate substantial opportunity to continue to improve PrEP offer rates. Our intervention delivered outreach to all women with a positive syphilis test result, including the 20% of the sample ultimately diagnosed with late latent or previously treated syphilis, and therefore likely overestimated PrEP eligibility due to current sexual risk. However, our approach allowed intervention delivery by a nonclinical team member and, while the optimal rate of PrEP offers is not readily ascertained, 17.6% is clearly too low. That increased PrEP offers did not lead to increased acceptance in the sample overall also suggests that additional tailored strategies may be necessary to overcome PrEP stigma and patient concerns about PrEP initiation.

Our data also highlight a need to improve delivery of comprehensive infection screening. We found low rates of concurrent HIV testing (33.6%) with the positive STI result. Anecdotally, the low rate of concurrent testing did not seem to be due to very recent HIV screening, as over one-third of participants had no lifetime HIV testing on file. Low rates of concurrent HIV/STI testing at the OB/GYN, including visits for people who are not pregnant and are seen for gynecologic care as well as individuals seen for prenatal visits, may relate to more integration of vaginal and urine-based screening into routine visits. This approach relies less on laboratory-based testing, which would require phlebotomy services. Specialized women's health clinics are a critical touchpoint, and strategies to increase HIV/STI co-testing, including through EHR order sets and rapid point-of-care HIV testing, should be explored and evaluated [31].

Four women in the study were diagnosed with new HIV infections, including 1 diagnosed concurrently with chlamydia and another diagnosed with HIV after 2 prior chlamydia infections, raising concern about under-recognition of chlamydia as a risk factor for HIV in women in the population evaluated [32]. In July 2021, on the basis of the data presented and updated PrEP guidelines [33], an additional PrEP navigator was hired for outreach to women with chlamydia. Understanding the impact of the expanded intervention scope on PrEP offers, acceptance, and prescribing is an important next step in the work.

Our study includes several limitations. Due to our observational design, we cannot rule out the possibility that the increase in PrEP offers was due to factors outside of the intervention, including secular trends in PrEP visibility. Despite our robust sample, we were likely underpowered to detect differences in later stages of the PrEP cascade. Furthermore, EHR demographic data likely missed some transgender women. We also relied on EHR fields for race/ethnicity, which are subject to inaccuracies [34]. Our study overlapped with the COVID-19 pandemic, which deepened existing barriers to accessing screening and HIV prevention services [35]. Due to data availability, our pre-intervention period was shorter than our postintervention period. Additionally, due to staffing constraints, our capacity to perform outreach for all patients with a positive chlamydia result within the study period was limited. Perhaps most importantly, our study evaluated PrEP delivery only to women with a positive STI, a cohort representing a very small fraction of those who would benefit from PrEP and those recommended for PrEP in the most recent CDC guidelines [33]. While PrEP delivery to women with STI is a critical intervention, and one likely to improve equity in the PrEP care cascade, this approach should be one part of a comprehensive effort to improve PrEP access for all those who stand to benefit. This includes investing in diverse, culturally competent PrEP teams, addressing structural barriers including transportation, insurance coverage, and child care, and offering PrEP in primary, urgent care, and low-barrier settings where patients already access services.

Overall, an interdisciplinary, system-level protocol providing PrEP outreach to women with positive STI results was associated with a significant increase in PrEP offers to Black and Hispanic women. Among non-Hispanic Black women, the intervention also yielded gains in PrEP acceptance. Because the intervention did not rely on advanced informatics infrastructure, it may be readily implemented in other safety-net settings. The identification of 4 new cases of HIV in our sample underscores the critical importance of increasing both routine HIV screening and PrEP uptake in all clinical settings caring for women. Results provide a potential pathway for health systems seeking to increase PrEP offers to women who have been inequitably served by PrEP interventions to date.
